# Phenolic Composition and Bioactivity of *Lavandula pedunculata* (Mill.) Cav. Samples from Different Geographical Origin

**DOI:** 10.3390/molecules23051037

**Published:** 2018-04-28

**Authors:** Catarina L. Lopes, Eliana Pereira, Marina Soković, Ana Maria Carvalho, Ana Maria Barata, Violeta Lopes, Filomena Rocha, Ricardo C. Calhelha, Lillian Barros, Isabel C.F.R. Ferreira

**Affiliations:** 1 Centro de Investigação de Montanha (CIMO), Instituto Politécnico de Bragança, Campus de Santa Apolónia, 5300-253 Bragança, Portugal; catarina.sp.lopes@alunos.ipb.pt (C.L.L.); eliana@ipb.pt (E.P.); anacarv@ipb.pt (A.M.C.); calhelha@ipb.pt (R.C.C.); lillian@ipb.pt (L.B.); 2 Institute for Biological Research “Siniša Stanković”, Department of Plant Physiology, University of Belgrade, Bulevar Despota Stefana 142, 11000 Belgrade, Serbia; mris@ibiss.bg.ac.rs; 3 Banco Português de Germoplasma Vegetal, Instituto Nacional de Investigação Agrária e Veterinária, I.P. (INIAV, I.P.), Quinta S. José, S. Pedro de Merelim, 4700-859 Braga, Portugal; ana.barata@iniav.pt (A.M.B.); violeta.lopes@iniav.pt (V.L.); filomena.rocha@iniav.pt (F.R.)

**Keywords:** phenolic composition, antioxidant activity, antimicrobial activity, antiproliferative effect, *Lavandula pedunculata* (Mill.) Cav

## Abstract

The aim of this study was to characterize the phenolic composition and evaluate the bioactivity of several samples of *Lavandula pedunculata* (Mill.) Cav, and to compare aqueous and hydroethanolic extracts. Plant materials were obtained by growing some accessions (seed samples) of various wild populations from different regions of Portugal conserved at the Portuguese Genebank in Braga. Phenolic compounds were analised by HPLC-DAD-ESI/MSn, antioxidant potential through in vitro assays (DPPH radical scavenging activity, reducing power and inhibition of lipid peroxidation), cytotoxicity on tumor cells (MCF-7, NCI-H460, HeLa and HepG2) and non-tumor (PLP2) cells, anti-inflammatory activity in rat RAW 264.7 macrophages, by the ability to inhibit NO production and antimicrobial potential by the microdilution method with INT dye (iodonitrotetrazolium chloride). Thirteen compounds were identified, being salvianolic acid B, rosmarinic acid and luteolin-7-*O*-glucuronide, the main compounds present, with values ranging between 44.3–582, 50.9–550, and 24.36–101.5 mg/g extract, respectively. *L. pedunculata* aqueous extract revealed a higher antioxidant potential (EC_50_ values between 14 to 530 μg/mL), which could be related to its higher concentration in phenolic compounds; however, the hydroethanolic extract showed a higher anti-inflammatory (lower EC_50_ values than 124 μg/mL) potential and antiproliferative capacity (lower GI_50_ values than 34 μg/mL). Thus, this study highlights the bioactive effects of this species and opens up possibilities of uses in food and pharmaceutical formulations. However, there are potential differences in such properties according to geographical origin of plant material, as in general, the samples from Alentejo presented higher results in all the bioactivities, compared with Trás-os-Montes samples.

## 1. Introduction

Bioactive compounds are molecules that have health benefits on living organisms, tissues or cells, being comprised of a wide range of classes, such as vitamins, carotenoids (lycopene, β-carotene and xanthophylls) and phenolic compounds [1]. These compounds are present in a wide range of foods (e.g., fruits, vegetables, plants and other food products) and its ingestion enables the induction of positive effects in the prevention and treatment of many human diseases, such as anxiety, insomnia, anorexia, cough and bronchitis [2].

Throughout the years, phenolic compounds have been largely studied in several foods, namely edible plants, due to their therapeutic capabilities in pathologies related to the nervous and gastrointestinal systems [3]; they also exhibit antioxidant, antitumor, anti-inflammatory and antiviral properties [1]. These molecules are characterized by being a very heterogeneous group of a great variety of compounds, such as phenolic acids, coumarins, flavonoids, stilbenes, hydrolysable and condensed tannins, lignans and lignins [3,4]. Flavonoids are one of the most abundant groups within phenolic compounds; their main mechanism of action goes through their ability of capturing reactive oxygen species and chelate metal ions, having a distinct antioxidant action [4]. Thus, the ingestion of antioxidants can significantly control the severity of chronic diseases, providing a close relationship between the uptake of free radicals and the involvement of endocrine responses. Therefore, the inclusion of antioxidants in the diet for the prevention of chronic diseases and for an improvement of general health, has been an important research target in recent decades [5].

*Lavandula pedunculata* (Mill.) Cav. (common English name French lavender and known in Portugal as rosmaninho, arçã, rosmaninho maior or lavanda), is a species belonging to the Lamiaceae which is native to the Iberian Peninsula, North Africa and Turkey [6,7,8]. In the Iberian Peninsula, this species has a widespread distribution and significant ethnobotanical uses documented [6,9]. It is considered the most resistant of all species of the *Lavandula* genus, well adapted to continental climates, especially to annual variation in temperature (hot summers and cold winters), growing in altitudes up to 1700 m [6,9]. In the context of traditional medicine, *L. pedunculata* has been used in infusions for internal and external applications, mainly recommended for the respiratory and digestive systems and as a therapeutic agent with antiseptic action for cleaning wounds [6,10].

Wild plant genetic resources, particularly of medicinal and aromatic species, have received increased interested worldwide. Besides *L. pedunculata* traits and potential use in different domains (e.g., as source of bioactive compounds), this endemic species had poor representation in germplasm collections and has not been comprehensively characterized [8]. Therefore, the Portuguese Genebank (BPGV) carried out in 2009, several collecting missions of *Lavandula* germoplasm in different regions of the national territory, including *L. pedunculata* [8]. The main purpose was to collect plant diversity, promoting ex situ conservation and providing morphological, molecular, chemical, and biochemical information (germplasm characterization); the seeds of several wild populations (genebank accessions) were stored at the BPGV facilities, in Braga, Portugal [8].

As bioactive molecules are often produced in response to changes in the surrounding environment, the same plant species growing at different sites may have different concentrations of the most promising compounds, or even different compounds in their constitution [11]. Germplasm accessions conserved at genebanks might generate very good matrices to evaluate useful characteristics and bioactive properties, because they correspond to stored plant materials of the same species but of different provenance. Moreover, genebanks procedures, such as germplasm characterization, provide the description of plant germplasm and determine the expression of highly heritable characters ranging from morphological, agronomical, chemical features to bioactive properties or molecular markers.

The aim of the present study was to evaluate the phenolic profile of the hydroethanolic and aqueous extracts of thirteen *Lavandula pedunculata* (Mill.) Cav. samples, whose seeds originated from different regions of Portugal, and are conserved ex-situ in the Portuguese Genebank. The bioactive properties were explored in terms of antioxidant, anti-inflammatory, cytotoxicity and antimicrobial potential. Furthermore, the study also addressed *L. pedunculata* germoplasm characterization (e.g., chemical and biochemical characteristics), contributing with relevant data about the studied accessions, enabling eventual discrimination among them, resulting in better insight about the composition of the germplasm collection and its genetic diversity.

## 2. Results

### 2.1. Phenolic Compounds

**Figure 1:**
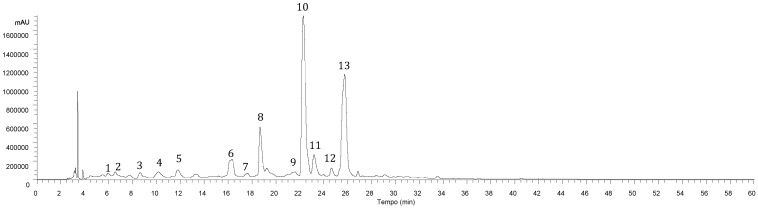
Chromatographic profile of sample 3 (Vila Viçosa, Évora) recorded at 280 nm, the peak numbers correspond to the compounds listed in Table 1.

Data related to the phenolic compounds identification (retention time, λmax in the visible region, molecular ion, main fragment ions in MS^2^, and tentative identification) obtained by HPLC-DAD-ESI/MSn analysis of *Lavandula pedunculata* (Mill.) Cav. samples are presented in Table 1. All samples presented a similar profile (Figure 1), revealing the presence of thirteen phenolic compounds, in both hydroethanolic and aqueous extracts, being nine identified as phenolic acids (mainly caffeic acid dimers, trimers and tetramers) and four flavonoids (mainly luteolin and eriodictyol glycoside derivatives).

Caffeic acid (compound **4**), luteolin-7-*O*-glucuronide (compound **8**) and rosmarinic acid (compound **10**) were positively identified by comparison with commercial standards. Rosmarinic acid has been one of the main compounds previously identified in *Lavandula* species [3,12,13,14,15]. Compounds **1** and **2**, **3** and **7** were identified as caffeic, *p*-coumaric and rosmarinic acid hexosides, based on the respective fragment ions released at *m*/*z* 179 [caffeic acid-H]^−^, 163 [coumaric acid-H]^−^ and 359 [rosmarinic acid-H]^−^ after loss of a hexosyl moiety (−162 mu). With the exception of rosmarinic acid hexoside, the mentioned compounds have been found in *Lavandula x intermedia Emeric* ex Loiseleur) Waste [12].

Compound **9** ([M − H]^−^ at *m*/*z* 719) released a main MS^2^ fragment at *m*/*z* 359 ([M − 2H]^2−^, rosmarinic acid), which allowed its identification as sagerinic acid [16]. Compound **11** ([M − H]^−^ at *m*/*z* 537) presented a similar UV spectrum and fragmentation pattern, consistent with the caffeic acid trimer lithospermic acid A [16,17,18]. Other identities, with the same molecular weight (salvianolic acids H/I), were discarded because they present quite a different fragmentation pattern [18,19]. Thus, being assigned to lithospermic acid A. Compound **13** ([M − H]^−^ at *m*/*z* 717) presented a fragmentation pattern with successive losses of 198 mu (danshensu) or 180 mu (caffeic acid) units, coherent with salvianolic acid B (also known as lithospermic acid B) [18,19]. These compounds have not been previously identified in *Lavandula* species, to the best of the authors’ knowledge.

The remaining compounds correspond to glycosylated flavones. Compound **5** ([M − H]^−^ at *m*/*z* 623) MS^2^ fragments revealed the alternative loss of hexosyl (*m*/*z* at 461; −162 u) and glucuronyl (*m*/*z* at 285; −176 u) residues, indicating location of each residue on different positions of the aglycone. No information about the identity of the sugar moieties and location on the aglycone could be obtained, so, the compound was identified as luteolin-*O*-hexosyl-*O*-glucuronide [20]. Compound **6** ([M − H]^−^ at *m*/*z* 463) was identified as a eriodictyol derivative, due to its UV-Vis spectra and MS fragmentation. This peak revealed the loss of glucuronyl (−176 u), therefore being assigned as eriodictyol-*O*-glucuronide [21]. Compound **12** ([M − H]^−^ at *m*/*z* 623) presented 42 u higher then compound 8, thus being assigned to methylluteolin-*O*-glucuronide [21]. Also, to the best of our knowledge, these compounds have not been previously identified in *Lavandula* species.

In a previous study performed by Costa et al. [3], using polar extracts from *Lavandula pedunculata* subsp. lusitanica, six different phenolic compounds were detected (3-*O*-caffeoylquinic acid, 4-*O*-caffeoylquinic acid, 5-*O*-caffeoylquinic acid, rosmarinic acid, luteolin and apigenin), nonetheless, only rosmarinic acid was the common phenolic acid identified, but revealed smaller amounts. However, also in this case, this compound proved to be the majority, in the hydroethanolic and ethanolic extracts. The differences observed between the different samples could be explained, due to the influence that the geographical area has on the chemical composition of plants, namely at the level of soil composition, climacteric environment, air humidity and daily sun exposure [11]. Moreover, to the best of the authors’ knowledge, and with the exception of the previous mentioned authors, there are no more reports describing the phenolic composition of *L. pedunculata* (Mill.) Cav.

Phenolic acids represent a significant part of this phenolic profile (Table 2), highlighting salvianolic acid B (compound **13**) and rosmarinic acid (compound **10**) as the major phenolic compounds present in both types of extracts. Regarding flavonoids, luteolin-7-*O*-glucuronide (compound **8**) was the main compound present (Table 2). In general, the aqueous extract revealed the highest content in phenolic compounds, revealing sample 8 (Ponte de Sôr, Portalegre) the highest concentration in total phenolic acids, flavonoids and total phenolic compounds for both the studied extracts. This difference could be explained to the chemical composition that each of the seeds initially presented and due to their different geographic origin.

### 2.2. Antioxidant Potential

The antioxidant activity of *L. pedunculata* hydroethanolic and aqueous extracts was evaluated using different in vitro assays (DPPH radical-scavenging activity, reducing power, inhibition of β-carotene bleaching and inhibition of lipid peroxidation—TBARS) and the results are present in Table 3. All samples, for both of the studied extracts, revealed antioxidant potential, EC_50_ values between 530–14 μg/mL and 1833–17 μg/mL for aqueous and hydroethanolic extract, respectively. In general, there is not a large divergence regarding EC_50_ values between the hydroethanolic and aqueous extracts. Accordingly, for aqueous extracts, sample 4 (Bragança) revealed the highest DPPH scavenging activity (EC_50_ = 68.0 μg/mL), while sample 2 (Évora) revealed the highest reducing power (EC_50_ = 51 μg/mL). For the β-carotene bleaching inhibition assay, samples 2 (Évora) and 10 (Castelo de Vide, Portalegre), with values of 253 and 236 μg/mL, respectively, revealed the highest potential. On the other hand, sample 1 (Marvão, Portalegre) revealed the highest antioxidant potential for the TBARS inhibition assay (14 μg/mL).

Otherwise, hydroethanolic extracts of samples 10 (Castelo de Vide, Portalegre), 11 (Elvas, Portalegre), 12 (Castelo de Vide, Portalegre), and 13 (Bragança) showed the lowest EC_50_ values for the β-carotene bleaching inhibition assay, presenting values of 252, 190, 223 and 214 μg/mL, respectively. Additionally, in the hydroethanolic extracts, samples 8 (Ponte de Sôr, Portalegre), 9 (Évora), and 2 (Évora) showed the highest antioxidant capacity for the DPPH scavenging activity, reducing power and TBARS inhibition assays, respectively. The highest EC_50_ values (lowest antioxidant activity) were detected in sample 13 (Bragança) for DPPH scavenging activity (257 μg/mL) and reducing power (216 μg/mL), in sample 8 for β-carotene bleaching inhibition (1833 μg/mL), and in samples 6 (Portalegre) and 11 (Elvas, Portalegre) in TBARS inhibition (63.5 and 62 μg/mL, respectively).

These results are in agreement with those found by Costa et al. [3], where the antioxidant potential *L. pedunculata* was evaluated by TBARS assay and at the highest concentration tested (5 mg/mL), the infusion, water and water:ethanol extracts completely prevented MDA production.

In a study performed by Pereira et al. [15], the antioxidant activity of different extracts (n-hexane, dichloromethane, ethyl acetate, methanol and water) of *L. pedunculata* was evaluated regarding the lipid peroxidation index (%), obtaining values that ranged from 26 to 41%. Moreover, Ferreira et al. [23] tested the ethanolic extracts and decoctions of *L. pedunculata* using the DPPH and β-carotene inhibition methods, in which these samples revealed antioxidant activity, presenting values between 93 and 20 mg/mL, respectively. Baptista et al. [24], also studied the antioxidant potential for the essential oils and extracts of two native Portuguese *Lavandula* species (*L. stoechas* subsp. luisieri and *L. pedunculata*) and considering the results obtained, there is some similarity in the results of the methanolic and aqueous extract, in comparison with the concentrations obtained in our study.

### 2.3. Anti-Inflammatory Activities

The in vitro anti-inflammatory results are present in Table 3. In general, hydroethanolic extracts revealed more promising anti-inflammatory potential (EC_50_ values ranging from 216 to 124 μg/mL), showing lower EC_50_ values than the aqueous extracts (EC_50_ values ranging between 140 to 301 μg/mL). Moreover, there were several samples that did not show anti-inflammatory activity (EC_50_ > 400 μg/mL), being the absence of this bioactivity higher for the aqueous samples. The samples that presented this activity were samples 4 (Bragança), 5 (Arronches. Portalegre), 7 (Nisa, Portalegre), 8 (Ponte de Sôr, Portalegre), 9 (Évora), and 13 (Bragança) in hydroethanolic extract, and samples 1 (Marvão, Portalegre), 2 (Évora), 3 (Vila Viçosa, Évora), 6 (Portalegre), 9 (Évora), 10 (Castelo de Vide, Portalegre), 11 (Elvas, Portalegre), 12 (Castelo de Vide, Portalegre), and 13 (Bragança) for the aqueous extracts.

Sample 6 hydroethanolic extract (Portalegre) presented the highest anti-inflammatory potential (124 μg/mL), while for the aqueous extract, sample 8 (Ponte de Sôr, Portalegre) revealed the highest efficiency, presenting a concentration of 140 μg/mL. Similarly, Algieri et al. [25] studied the anti-inflammatory activity of *L. stoechas* hydromethanolic extract, which also exhibited a significantly inhibition of carrageenan-induced paw edema in mice. Therefore, the hydroethanolic extracts could also be considered as a good source of anti-inflammatory molecules.

### 2.4. Cytotoxicity

Table 4 summarizes the effects of *L. pedunculata* hydroethanolic and aqueous extracts (infusions) on the inhibition of the growth of four human tumor cell lines (MCF-7, NCI-H460, HeLa and HepG2). In this study, it was evident the cytotoxic activity of *L. pedunculata* in all the cell lines tested, ranging values between 374–82 μg/mL and 340–34 μg/mL, for aqueous and hydroethanolic extracts, respectively. Comparing the two types of studied extracts, the aqueous extracts showed anti-proliferative potential in more samples and in more cell lines, however it was the hydroethanolic extract that, overall, showed a higher cytotoxic potential, i.e., lower GI_50_ values (34 μg/mL). Sample 6 (Portalegre) hydroethanolic extract presented the best cytotoxic potential in MCF-7 (values ranged from 53 to 236 μg/mL) and HepG2 (values between from 34 and 212 μg/mL) cell lines. On the other hand, sample 12 (Castelo de Vide, Portalegre) showed a higher antiproliferative capacity in NCI-H460 cell line (GI_50_ values = 119 μg/mL); and sample 10 (Castelo de Vide, Portalegre) showed a lower GI_50_ value in the HeLa cell line (62 μg/mL). In hydroethanolic extract, sample 6, 12 and 13 (Bragança) revealed lower antiproliferative capacity in NCI-H460 (340 μg/mL), MCF-7 (236 μg/mL) and HeLa (222 μg/mL) cell lines, respectively; in HepG2 cell line were samples 7 (Nisa, Portalegre) and 13 that evidenced a lower, with GI_50_ values of 212 and 204 μg/mL, respectively.

On the other hand, the aqueous extract of sample 9 (Évora) revealed a better cytotoxic potential in MCF-7 and HepG2 cell lines, with values of 150 and 118 μg/mL, respectively. In the NCI-H460 cell line, the values ranged from 374 and 113 μg/mL, highlighting sample 4 (Bragança) with the highest antiproliferative activity. Meanwhile, for HeLa cell line, sample 12 (Castelo de Vide, Portalegre) showed the lowest GI_50_ value (82 μg/mL).

The cytotoxicity in non-tumor cells were tested using a primary culture cell line obtained from porcine liver (PLP2 cell culture) and was used as a preliminary toxicity model for normal cells. None of the samples showed toxicity (GI_50_ > 400 μg/mL), with the exception of hydroethanolic extract of sample 12 (Castelo de Vide, Portalegre), and aqueous extracts of sample 7 (Nisa, Portalegre), 8 (Ponte de Sôr, Portalegre) and 9 (Évora). However, the presented concentrations are much higher than those obtained for the antiproliferative inhibition using the studied tumor cell lines.

Despite being the same plant species, it is normal the existence of different results. The geographical origin of the seeds is one of the factors that can directly influence the chemical and nutritional composition of plants due to biotic and abiotic factors [11], thus, in this case, as the seeds were harvested from different areas, this factor could lead to the observed oscillations in the bioactive potential.

There are few cytotoxicity studies carried out using this species, however the results described in literature revealed lower cytotoxic potential, when compared to our results. According to Pereira et al. [15], the results obtained with *L. pedunculata* methanolic extracts suggest that for concentrations up to 15 µg/mL and an incubation period of 24 h, no relevant cytotoxic effects were observed. Tang et al. [26], tested five human tumor cell lines (NB4, A549, SHSY5Y, PC3, and MCF7) using the methanolic extract of *L. angustifolia*, showed a weak inhibitory activity against the tested human tumor cell lines (IC_50_ values ranging from 2.2–8.2 mM).

### 2.5. Antimicrobial Activity

The results on antibacterial and antifungal activities of *L. pedunculata* hydroethanolic and aqueous extracts are presented in Table 5. The samples were tested against a panel of eight bacteria and fungi strains, specifically selected on the basis of their importance to public health.

For antibacterial activity of the hydroethanolic extracts, samples 1 (Marvão, Portalegre) and 5 (Arronches, Portalegre) revealed a higher activity (lower MICs) for all the studied strains. In these cases, MIC values range from 20 µg/mL in sample 1 for the *Staphylococcus aureus* strain and 40 µg/mL in sample 5 for the *Enterobacter cloacae* strain. The lowest MBC values were observed for samples 1 and 3 (Vila Viçosa, Évora) in the inhibition of *Staphylococcus aureus* strain (40 µg/mL). In the evaluation of *L. pedunculata* aqueous extracts, sample 5 showed the lowest MIC and MBC, being more potent for *Bacillus cereus* strain (MIC = 75 µg/mL and MBC = 150 µg/mL), along with the MBC of sample 8 (Ponte de Sôr, Portalegre) in *Escherichia coli* strain (also 150 µg/mL). These results are in agreement with previous studies, where other plants species also showed antimicrobial activity: for example, *Castanea sativa* Mill. flowers [27], *Alnus rugosa* L. aerial parts [28], and *Veronica urticifolia* Jacq. [29]. Nikolic et al. [30] studied the essential oils of different *Thymus* species, such as *T. serpyllum*, *T. algeriensis* and *T. vulgaris*, which showed antimicrobial activity. However, to the best of the author’s’ knowledge there are no studies reporting the antibacterial activity of the studied *Lavandula* species.

Considering the antifungal activity, for hydroethanolic extracts, all samples revealed inhibitory and fungicidal activity. Sample 3 showed the best MIC values for all the tested strains, with the exception of *Aspergillus versicolor* and *Aspergillus ochraceus*, where samples 2 and 9 revealed the highest potential, respectively. Regarding the MFC, in general, most of the samples revealed promising results, being better for sample 13 aqueous extract. In the case of aqueous extracts, only a few samples showed activity in all the studied strains, meanwhile samples 2, 11, 12 and 13 showed inhibitory and fungicidal potential for all the studied fungi strains. Samples 9 and 11 presented the highest inhibitory capacity for all the fungal strains analysed. The best fungicide capacity was evident in sample 11 against *Aspergillus versicolor* strain.

The antifungal potential is, also, in agreement with other studies regarding antifungal activity of other plant species, which were carried out by other authors, such as *Castanea sativa* [27] and *Alnus rugosa* [28]. Once more, to the best of the authors’ knowledge, there are no previous studies on the antifungal activity of *L. pedunculata*.

## 3. Materials and Methods

### 3.1. Samples and Samples Preparation

The studied samples concern plant materials (the top 20 cm of the flowering stems with inflorescences) randomly harvested in each of thirteen field plots of *Lavandula pedunculata* (Mill.) Cav. (botanical family Lamiaceae) grown at the Portuguese Genebank (Banco Português de Germoplasma Vegetal, BPGV).

The Portuguese Genebank conserves ex situ, using cold temperatures (e.g., 5–6 °C), a collection of medicinal and aromatic plants, including species of the genus *Lavandula*. The col, lection consists of seed samples (accessions) of wild specimens that have been randomly collected in different natural populations within several Portuguese regions [8].

For this study, the stored seeds of thirteen accessions of *L. pedunculata,* of the BPGV collection were sown and the plantlets transplanted outdoor to thirteen field plots, in BPGV farm at S. Pedro de Merelim, Braga, Northern Portugal (GPS coordinates 41°34’28.01’’ N; 8°27’09.21’’ S).

In 2015, the flowering parts in blossom of each accession grown were harvested, kept in paper bags at −20 °C, corresponding individually to a sample. Each sample of plant material (approximately 200 g) was subsequently lyophilized (FreeZone 4.5, Labconco, Kansas City, MO, USA), reduced to a fine dried powder (~20 mesh) and mixed to obtain a homogenate sample, corresponding to a total of thirteen different samples.

The thirteen studied accessions of *L. pedunculata* were gathered wild in two Portuguese regions, Alentejo and Trás-os-Montes (Table 6), altitude range between 100 and 1000 m [8]. This species has several synonymies such as *Lavandula eliasii* Sennen, *Lavandula pedunculata* subsp. *pedunculata*, *Lavandula stoechas* subsp. *pedunculata* (Mill.) Samp. ex Rozeira, *Lavandula stoechas* subsp. *lusitanica* (Chaytor) Rozeira, and *Stoechas pedunculata* Mill. [9].

The hydroethanolic extracts were obtained from the lyophilized plant material. The dried sample was extracted following a procedure previously described by Pereira et al. [31]. A solution of ethanol/water (80:20 *v*/*v*) (25 mL) was added to 1 g of sample and stirring for 1 h (25 °C at 150 rpm) and subsequently filtered through Whatman No. 4 paper. The residue was then re-extracted with an additional portion of 25 mL of the same solution (25 °C at 150 rpm) during 1 h. The combined extracts were evaporated to remove the ethanolic fraction (at 40 °C) and then the water was frozen and lyophilized.

The aqueous extracts (infusions) preparation was established according a study performed by Pereira et al. [31]. Powdered samples (2 g) were added to 200 mL of boiling distilled water, left to stand at room temperature for 5 min, and then filtered under reduced pressure, frozen and lyophilized.

### 3.2. Standards and Reagents

Acetonitrile 99.9% was of HPLC grade from Fisher Scientific (Lisbon, Portugal). Phenolic compound standards (caffeic acid ≥ 99%, *p*-coumaric acid ≥ 90%; hesperetin ≥ 99%, luteolin-7-*O*-glucoside ≥ 99%, quercetin-3-*O*-glucoside ≥ 99%, rosmarinic acid ≥ 99% HPLC purity) were from Extrasynthese (Genay, France). Trypan blue, lipopolysaccharide (LPS), dexamethasone, acetic acid, formic acid, ellipticine, sulforhodamine B (SRB), trichloroacetic acid (TCA) and Tris were all purchased from Sigma-Aldrich (St. Louis, MO, USA). Dulbecco’s modified Eagle’s medium was purchased from HyClone. RAW264.7 cells were acquired from ECACC (‘‘European Collection of Animal Cell Culture”) (Salisburg, UK), Griess reagent system kit was purchased from Promega, Fetal bovine serum (FBS), L-glutamine, Hank’s balanced salt solution (HBSS), trypsin-EDTA (ethylenediaminetetraacetic acid), penicillin/streptomycin solution (100 U/mL and 100 mg/mL, respectively), RPMI-1640 and DMEM media were from Hyclone (Logan, UT, USA). Water was treated in Milli-Q water purification system (TGI Pure Water Systems, Greenville, SC, USA).

### 3.3. Analysis of Phenolic Compounds

The lyophilized extracts were re-dissolved at 5 mg/mL in ethanol/water (20:80, *v*/*v*) and 100% water for hydroethanolic and aqueous extract, respectively, and filtered through a 0.22-µm disposable LC filter disk. Chromatographic analyses were performed in a Dionex Ultimate 3000 UPLC (Thermo Scientific, San Jose, CA, USA) system equipped with a diode array detector (DAD) coupled to an electrospray ionization mass detector (LC-DAD-ESI/MSn) as previously described by Bessada et al. [32].

Chromatographic separation was achieved with a Waters Spherisorb S3 ODS-2 C18 (3 μm, 4.6 mm × 150 mm, Waters, Milford, MA, USA) column thermostatted at 35 °C. The solvents used were: (A) 0.1% formic acid in water; (B) acetonitrile (HPLC purity). The elution gradient established was isocratic 15% B (5 min), 15% B to 20% B (5 min), 20-25% B (10 min), 25–35% B (10 min), 35–50% B (10 min), and re-equilibration of the column, using a flow rate of 0.5 mL/min. Double online detection was carried out in the DAD using 280, 330, 370 and 520 nm as preferred wavelengths and in a mass spectrometer (MS) connected to HPLC system via the DAD cell outlet.

MS detection was performed in negative mode, using a Linear Ion Trap LTQ XL mass spectrometer (Thermo Finnigan, San Jose, CA, USA) equipped with an ESI source. Nitrogen served as the sheath gas (50 psi); the system was operated with a spray voltage of 5 kV, a source temperature of 325 °C, a capillary voltage of −20 V. The tube lens offset was kept at a voltage of −66 V. The full scan covered the mass range from *m*/*z* 100 to 1500. The collision energy used was 35 (arbitrary units). Data acquisition was carried out with Xcalibur^®^ data system (Thermo Finnigan, San Jose, CA, USA).

Compounds were carefully identified comparing the obtained information: retention times, UV-vis and mass spectra, with those obtained from standard compounds, when available. Otherwise, compounds were tentatively identified comparing the obtained information with available data reported in the literature. For the quantification, a calibration curve was constructed for each available phenolic standard based on the UV signal (maximum absorption of each standard compound lambda max). When a commercial standard was not available, the quantification was performed through the calibration curve of the most similar available standard. The results were expressed as mg/g of extract.

### 3.4. Evaluation of the Antioxidant Activity

The lyophilized extracts were re-dissolved in ethanol/water (80:20 *v*/*v*) and water for hydroethanolic and aqueous extracts, respectively (final concentration 10 mg/mL), and submitted to various in vitro colorimetric assays (DPPH radical-scavenging activity, reducing power, inhibition of β-carotene bleaching and inhibition of lipid peroxidation—TBARS).

DPPH radical-scavenging activity was performed using an ELX800 Microplate Reader (Bio-Tek). The reaction mixture in each one of the 96-wells consisted of one of the different concentrations of the extracts (30 µL) and the methanolic solution containing DPPH radicals (6 × 10^−5^ mol/L, 270 µL). The mixture was left to stand for 60 min in the dark. The reduction of the DPPH radical was determined by measuring the absorption at 515 nm. The radical scavenging activity (RSA) was calculated by the equation: *% RSA = [(A_DPPH_ − A_S_)*/*A_DPPH_] × 100* (A_S_ is the absorbance of the solution when the sample extract has been added at a particular level and A_DPPH_ is the absorbance of the DPPH solution). The extract concentration providing 50% of the radicals scavenging activity (EC_50_) was calculated from the graph of RSA percentage against extract concentration.

Reducing power evaluation was performed using the Microplate Reader cited above. The different concentrations of the extracts (0.5 mL) were mixed with sodium phosphate buffer (200 mmol/L, pH 6.6, 0.5 mL) and potassium ferricyanide (1% *w*/*v*, 0.5 mL). For each concentration, the mixture was incubated at 50 °C during 20 min, and then was added trichloroacetic acid (10% *w*/*v*, 0.5 mL). The mixture (0.8 mL) was poured in the 48-wells, as were deionized water (0.8 mL) and ferric chloride (0.1% *w*/*v*, 0.16 mL), and was measured the absorbance at 690 nm. The extract concentration providing 0.5 of absorbance (EC_50_) was calculated from the graph of absorbance at 690 nm against extract concentrations.

In inhibition of β-carotene bleaching, a solution of β-carotene was previously prepared dissolving β-carotene (2 mg) in chloroform (10 mL) and 2 mL of this solution were pipetted into a round-bottom flask. After chloroform removed (at 40 °C under vacuum) linoleic acid (40 mg), Tween 80 emulsifier (400 mg), and distilled water (100 mL) were added with vigorous shaking. Aliquots (4.8 mL) of this emulsion were transferred into different tubes containing different concentrations of the extracts (0.2 mL). The tubes were shaken and incubated (at 50 °C) in a water bath. As soon as the emulsion was added to each tube, the zero-time absorbance was measured at 470 nm. β-carotene bleaching inhibition was calculated using the equation: *β-carotene content after 2 h of assay*/*initial β-carotene content) × 100*. The extract concentration providing 50% antioxidant activity (EC_50_) was calculated by interpolation from the graph of β-carotene bleaching inhibition percentage against extract concentration.

Lipid peroxidation inhibition was evaluated using a porcine (Sus scrofa) brains (obtained from official slaugh-tered animals). This is homogenized with Polytron in an ice-cold Tris–HCl buffer (20 mM, pH 7.4) to produce a 1:2, *w*/*v* brain tissue homogenate which was centrifuged (3000× *g*; 10 min). Analiquot (100 μL) of the supernatant was incubated with the sample solutions (200 μL) in the presence of FeSO_4_ (10 mM; 100 μL) and ascorbic acid (0.1 mM; 100 μL) at 37 °C during 1 h. The reaction was stopped by the addition of trichloroacetic acid (28%, *w*/*v*, 500 μL), and then thiobarbituric acid (TBA, 2%, *w*/*v*, 380 μL). The mixture was then heated at 80 °C during 20 min. After centrifugation (3000× *g*; 10 min), the color intensity of the malondialdehyde (MDA)–TBA complex in the supernatant was measured (at 532 nm). The inhibition ratio (%) was calculated using the formula: *inhibition ratio (%) = [(A − B)*/*A] × 100%* (A were the absorbance of the control and B were the absorbance of sample solution). The extract concentration providing 50% of antioxidant activity (EC_50_) was calculated from the graph of TBARS formation inhibition against extract concentrations.

All the results were expressed in EC_50_ values (µg/mL) and trolox was used as positive control [33].

### 3.5. Evaluation of the Cytotoxicity

#### 3.5.1. General

For cytotoxicity evaluation all lyophilized extracts were re-dissolved in water, with a final solution of 8 mg/mL and diluted to different concentrations. The assay was performed according a procedure described by Guimarães et al. [34]. Ellipticine was used as positive control and the results were calculated as GI_50_ values (sample concentration that inhibited 50% of the net cell growth).

#### 3.5.2. In Tumor Cell Lines

Four human tumor cell lines were used to acee the cytotoxicity evaluation: MCF-7 (breast adenocarcinoma), NCI-H460 (non-small cell lung cancer), HeLa (cervical carcinoma) and HepG2 (hepatocellular carcinoma). Cells were routinely maintained as adherent cell cultures in RPMI-1640 medium containing 10% heat-inactivated FBS (MCF-7, NCI-H460 and HCT-15) and 2 mM glutamine or in DMEM supplemented with 10% FBS, 2 mM glutamine, 100 U/mL penicillin and 100 mg/mL streptomycin (HeLa and HepG2 cells), at 37 °C, in a humidified air incubator containing 5% CO_2_. The cell lines were plated an appropriate density (1.0 × 10^4^ cells/well) in 96-well plates and allowed to attach for 24 h. Cells were then treated for 48 h with several extract concentrations. Following this incubation period, the adherent cells were fixed by adding cold 10% trichloroacetic acid (TCA, 100 μL) and incubated during 60 min at 4 °C. Plates were then washed with deionized water and dried; sulforhodamine B solution (0.1% in 1% acetic acid, 100 μL) was then added to each plate well and incubated during 30 min at room temperature. Unbound SRB was removed by washing with 1% acetic acid. Plates were air-dried, the bound SRB was solubilized with 10 mM Tris (200 μL) and the absorbance was measured at 540 nm in the microplate reader cited above. The results were expressed in GI_50_ values; sample concentration that inhibited 50% of the net cell growth [34].

#### 3.5.3. In Non-Tumor Cells

For the hepatotoxicity evaluation, a cell culture was prepared from a freshly harvested porcine liver obtained from a local slaughter house, and it was designed as PLP2. Briefly, the liver tissues were rinsed in hank’s balanced salt solution containing 100 U/mL penicillin, 100 µg/mL streptomycin and divided into 1 × 1 mm^3^ explants. Some of these explants were placed in 25 cm^2^ tissue flasks in DMEM medium supplemented with 10% fetal bovine serum, 2 mM nonessential amino acids and 100 U/mL penicillin, 100 mg/mL streptomycin and incubated at 37 °C with a humidified atmosphere containing 5% CO_2_. The medium was changed every two days. Cultivation of the cells was continued with direct monitoring every two to three days using a phase contrast microscope. Before confluence was reached, cells were subcultured and plated in 96-well plates at a density of 1.0 × 10^4^ cells/well, and cultivated in DMEM medium with 10% FBS, 100 U/mL penicillin and 100 µg/mL streptomycin [34].

### 3.6. Evaluation of the Anti-Inflammatory Activity

For the cells treatment, the mouse macrophage-like cell line RAW 264.7 was cultured in DMEM medium supplemented with 10% heat-inactivated fetal bovine serum, glutamine and antibiotics at 37 °C under 5% CO_2_, in humidified air. For each experiment, cells were detached with a cell scraper. In the experiment cell density of 5 × 10^5^ cells/mL was used, and the proportion of dead cells was less than 5% according to the Trypan blue dye exclusion test. Cells were seeded in 96-well plates at 150,000 cells/well and allowed do attach to the plate overnight. Subsequently, cells were treated with the several concentrations of each extract during 1 h. Dexamethasone (50 µM) was used as a positive control for the experiment. The following step was the stimulation with LPS (1 µg/mL) for 18 h. The effect of all the tested samples in the absence of LPS was also analyzed, to observe if they induced changes in Nitric oxide (NO) basal levels. In negative controls, no LPS was added. Both extracts and LPS were dissolved in supplemented DMEM.

Anti-inflammatory assay was performed in concentration range 400–125 μg/mL and dexamethasone (50 μM) was used as a positive control. The mouse macrophage-like cell line RAW 264.7 stimulated with LPS was used in the assay. Nitric oxide (NO) production was studied with Griess Reagent System kit. Results were expressed as EC_50_ values (μg/mL) equal to the sample concentration providing a 50% inhibition of NO production [35].

### 3.7. Evaluation of the Antimicrobial Activity

#### 3.7.1. Antibacterial Activity

*Escherichia coli* (ATCC (American type culture collection) 35210), *Enterobacter cloacae* (ATCC 35030), *Salmonella typhimurium* (ATCC 13311), and *Pseudomonas aeruginosa* (ATCC 27853), were the used Gram-negative bacteria used, while *Listeria monocytogenes* (NCTC (National collection of type cultures) 7973), *Micrococcus flavus* (ATCC 10240), *Bacillus cereus* (clinical isolate), *Staphylococcus aureus* (ATCC 6538), were the used Gram-positive bacteria. The minimum inhibitory (MIC) and minimum bactericidal (MBC) concentrations were determined by the microdilution method. Each fresh overnight culture of bacteria was adjusted spectrophotometrically (625 nm) to a concentration of 1 × 10^5^ CFU/mL. Dilutions of inocula were cultured on solid medium to verify the absence of contamination and check the validity of each inoculum. Different dilutions of the aqueous extract were added to the wells containing 100 μL of Tryptic Soy Broth (TSB) and afterwards, 10 μL of inoculum was added to all wells. The microplates were incubated for 24 h at 37 °C. The MIC of the samples was detected following the addition of 40 μL of iodonitrotetrazolium chloride (INT) (0.2 mg/mL) and incubation at 37 °C for 30 min. The lowest concentration that produced a significant inhibition (around 50%) of the growth of the bacteria in comparison with the positive control was identified as the MIC. MICs, obtained from the susceptibility testing of various bacteria to tested extracts were determined also by a colorimetric microbial viability assay based on the reduction of the INT colour and compared with a positive control for each bacterial strain. MBC were determined by serial sub-cultivation of 10 μL into microplates containing 100 μL of TSB. The lowest concentration that showed no growth after this sub-culturing was read as the MBC [36]. The results were expressed in mg/mL.

#### 3.7.2. Antifungal Activity

*Aspergillus ochraceus* (ATCC 12066), *Aspergillus versicolor* (ATCC 11730), *Aspergillus niger* (ATCC 6275), *Aspergillus fumigatus* (ATCC 1022), *Trichoderma viride* (IAM (Culture Collection, Center for Cellular and Molecular Research, Institute of Molecular and Cellular Biosciences, The University of Tokyo, Japan) 5061), *Penicillium funiculosum* (ATCC 36839), *Penicillium ochrochloron* (ATCC 9112) and *Penicillium verrucosum* var. *cyclopium* (food isolate) were used. Fungal spores were washed from the surface of agar plates with sterile 0.85% saline containing 0.1% Tween 80 (*v*/*v*). The spore suspension was adjusted with sterile saline to a concentration of approximately 1.0 × 10^5^ in a final volume of 100 μL per well. The inocula were stored at 4 °C for further use. Dilutions of each inoculum were cultured on solid MA to verify the absence of contamination and to check the validity of the inoculum. MIC determination was also performed by a serial dilution technique using 96-well microtitre plates. The investigated sample was dissolved in water and added to broth malt medium with a fungal inoculum. The microplates were incubated during 72 h at 28 °C. The lowest concentrations without visible growth (as assessed using a binocular microscope) were defined as the MICs. The minimum bactericidal and fungicidal concentrations (MBC and MFC) were determined by serial sub-cultivation of 2 μL in microtitre plates containing 100 μL of malt broth per well and further incubation for 72 h at 28 °C. The lowest concentration with no visible growth was defined as the MFC, indicating 99.5% killing of the original inoculum. Streptomycin and ampicillin, bifonazole and ketoconazole (in a range of 0.01 to 5 mg/mL) were used as positive controls and for negative control 5% DMSO was used [36]. The results were expressed in μg/mL.

### 3.8. Statistical Analysis

In this study all the assays were carried out in triplicate. The results are expressed as mean values and standard deviation (SD). All results were analyzed using one-way analysis of variance (ANOVA) followed by Tukey’s HSD Test with α = 0.05. These analyses were carried out using IBM SPSS Statistics, Version 23.0. (IBM Corp., Armonk, New York, USA).

## 4. Conclusions

Overall, the study of natural matrices allows a greater knowledge of their chemical composition and, therefore, a more adequate applicability. The present study allowed to deep knowledge on the phenolic profile of *L. pedunculata*, since samples with different origins were studied. In general, most of the samples revealed diverse bioactive properties, such as, cytotoxicity, antioxidant, anti-inflammatory and antimicrobial activity. The aqueous extracts showed a higher concentration of phenolic compounds and a higher antioxidant activity; nevertheless, the hydroethanolic extracts exhibited a higher anti-inflammatory potential in most of the samples, as also a higher antiproliferative capacity and antimicrobial activity. This may be due to the fact that hydroethanolic extracts contains other compounds (non-phenolic compounds), which could be correlated with these bioactivities.

Besides the great importance of *L. pedunculata* bioactive properties, these results also highlight the existing variances between samples from different geographical origin. Further studies are required to correlate biotic and abiotic factors with the chemical composition of the specimens.

The studied plant materials were obtained from previously conserved seeds (accessions) of different *L. pedunculata* populations, of the Portuguese Genebank collection. These outcomes contributed to data on important chemical characteristics, which might distinguish accessions within a species. Such characterization provides essential information assuring the best utilization of the conserved germplasm to the final users, meeting the goal of ex situ conservation of germplasm in genebanks.

## Figures and Tables

**Table 1:** Retention time (Rt), wavelengths of maximum absorption in the visible region (λ_max_), mass spectral data and tentative identification of phenolic compounds in *L. pedunculata* samples.

Peak	Rt (min)	λ_max_ (nm)	[M-H]^−^ (*m*/*z*)	MS^2^ (*m*/*z*)	Tentative Identification	Identification Type
1	5.97	320	341	179(100)	Caffeic acid hexoside	[12], DAD/MS
2	6.57	320	341	179(100)	Caffeic acid hexoside	[12], DAD/MS
3	8.65	310	325	163(100)	*p*-Coumaroyl hexoside	[12], DAD/MS
4	10.16	320	179	119(100)	Caffeic acid	Standard/DAD/MS
5	13.22	332	623	461(27), 285(100)	Luteolin-*O*-hexosyl-*O*-glucuronide	[20], DAD/MS
6	16.34	284,330 sh	463	287(100)	Eriodictyol-*O*-glucuronide	[21], DAD/MS
7	17.64	320	521	359(100), 197(8), 179(14), 161(3), 135(5)	Rosmarinic acid hexoside	[22], DAD/MS
8	18.68	347	461	285(100)	Luteolin-7-*O*-glucuronide	Standard/DAD/MS
9	19.23	283,338 sh	719	539(29), 521(18), 359(100), 179(32), 161(5), 135(5)	Sangerinic acid	[16], DAD/MS
10	22.29	325	359	197(27), 179(37), 161(100), 135(5)	Rosmarinic acid	Standard/DAD/MS
11	23.19	288,328 sh	537	493(18), 359(100), 313(11), 295(5), 197(3), 179(3)	Lithospermic acid A	[17,18,19], DAD/MS
12	24.65	340	475	299(100), 284(68)	Methylluteolin-*O*-glucuronide	[21], DAD/MS
13	25.75	309,338sh	717	537(47), 519(17), 493(40), 359(97), 339(10), 321(8), 313(17), 295(100), 197(7), 179(27)	Salvianolic acid B	[18,19], DAD/MS

**Table 2:** Phenolic compounds quantification (mg/g of extract, mean ± SD) in *L. pedunculata* hydroethanolic and aqueous extracts.

	1	2	3	4	5	6	7	8	9	10	11	12	13
**Hydroethanol Extract**													
**Peak** 1	0.28 ± 0.01 ^f^	0.034 ± 0.003 ^k^	0.179 ± 0.001 ^i^	tr	0.210 ± 0.005 ^h^	0.23 ± 0.01 ^g^	0.11 ± 0.04 ^j^	2.44 ± 0.02 ^a^	0.37 ± 0.01 ^d^	0.44 ± 0.01 ^c^	0.277 ± 0.004 ^f^	0.59 ± 0.02 ^b^	0.33 ± 0.02 ^e^
**Peak** 2	0.18 ± 0.02 ^h^	0.079 ± 0.003 ^j^	0.31 ± 0.001 ^d^	0.120 ± 0.003 ^i^	0.22 ± 0.02 ^g^	0.295 ± 0.001 ^e^	0.24 ± 0.01 ^f^	1.87 ± 0.02 ^a^	0.40 ± 0.01 ^c^	0.31 ± 0.02 ^de^	0.22 ± 0.01 ^fg^	0.54 ± 0.02 ^b^	0.115 ± 0.001 ^i^
**Peak** 3	0.77 ± 0.01 ^f^	1.002 ± 0.001 ^d^	0.80 ± 0.002 ^f^	0.50 ± 0.01 ^h^	0.95 ± 0.02 ^e^	0.98 ± 0.01 ^de^	1.48 ± 0.03 ^a^	1.27 ± 0.07 ^b^	1.16 ± 0.03 ^c^	0.98 ± 0.03 ^de^	0.80 ± 0.01 ^f^	1.26 ± 0.01 ^b^	0.57 ± 0.03 ^g^
**Peak** 4	0.63 ± 0.02 ^i^	0.805 ± 0.002 ^f^	0.88 ± 0.001 ^d^	0.639 ± 0.002 ^hi^	1.01 ± 0.02 ^c^	0.85 ± 0.02 ^e^	1.14 ± 0.02 ^b^	1.49 ± 0.03 ^a^	1.00 ± 0.04 ^c^	0.802 ± 0.001 ^f^	0.72 ± 0.03 ^g^	1.12 ± 0.04 ^b^	0.65 ± 0.02 ^h^
**Peak** 5	2.05 ± 0.03 ^f^	2.33 ± 0.03 ^c^	2.68 ± 0.01 ^b^	1.37 ± 0.01 ^k^	1.846 ± 0.001 ^g^	1.84 ± 0.01 ^g^	1.599 ± 0.004 ^i^	1.54 ± 0.01 ^j^	2.22 ± 0.01 ^d^	2.695 ± 0.003 ^a^	2.09 ± 0.01 ^e^	2.06 ± 0.01 ^f^	1.698 ± 0.003 ^h^
**Peak** 6	0.137 ± 0.003 ^h^	tr	0.40 ± 0.01 ^c^	0.74 ± 0.01 ^b^	0.082 ± 0.003 ^i^	tr	0.060 ± 0.001 ^j^	0.782 ± 0.003 ^a^	tr	0.299 ± 0.005 ^d^	0.150 ± 0.003 ^g^	0.231 ± 0.004 ^e^	0.17 ± 0.01 ^f^
**Peak** 7	1.18 ± 0.01 ^g^	1.379 ± 0.001 ^de^	1.52 ± 0.01 ^b^	0.93 ± 0.02 ^i^	1.40 ± 0.05 ^cd^	1.355 ± 0.004 ^ef^	1.39 ± 0.04 ^cde^	1.78 ± 0.06 ^a^	1.51 ± 0.09 ^b^	1.32 ± 0.01 ^f^	1.38 ± 0.05 ^de^	1.43 ± 0.03 ^c^	1.047 ± 0.002 ^h^
**Peak** 8	13.10 ± 0.01 ^l^	19.93 ± 0.02 ^e^	21.68 ± 0.03 ^c^	22.35 ± 0.06 ^b^	12.15 ± 0.02 ^m^	17.37 ± 0.02 ^i^	18.62 ± 0.01 ^g^	20.66 ± 0.01 ^d^	19.7 ± 0.1 ^f^	15.60 ± 0.02 ^k^	17.57 ± 0.01 ^h^	16.54 ± 0.02 ^j^	24.36 ± 0.04 ^a^
**Peak** 9	1.99 ± 0.01 ^e^	2.34 ± 0.05 ^c^	2.25 ± 0.04 ^d^	2.90 ± 0.01 ^a^	1.77 ± 0.08 ^g^	1.97 ± 0.01 ^ef^	2.38 ± 0.03 ^bc^	2.4 ± 0.1 ^b^	2.84 ± 0.08 ^a^	2.20 ± 0.05 ^d^	1.93 ± 0.01 ^f^	1.13 ± 0.04 ^i^	1.41 ± 0.03 ^h^
**Peak** 10	21.4 ± 0.40 ^j^	34.4 ± 0.4 ^e^	38.5 ± 0.60 ^b^	16.9 ± 0.5 ^k^	36.0 ± 0.2 ^c^	32.5 ± 0.6 ^f^	26.11 ± 0.05 ^h^	50.9 ± 0.9 ^a^	30.4 ± 0.4 ^g^	35.2 ± 0.6 ^d^	23.5 ± 0.2 ^i^	17.0 ± 0.6 ^k^	7.5 ± 0.2 ^l^
**Peak** 11	2.89 ± 0.01 ^k^	4.27 ± 0.06 ^f^	5.4 ± 0.4 ^c^	4.00 ± 0.09 ^gh^	3,52 ± 0.08 ^j^	3.546 ± 0.002 ^ij^	3.69 ± 0.09 ^i^	6.4 ± 0.1 ^a^	5.15 ± 0.04 ^d^	4.4 ± 0.1 ^e^	3.95 ± 0.1 ^h^	4.1 ± 0.6 ^fg^	6.0 ± 0.01 ^b^
**Peak** 12	2.520 ± 0.001 ^i^	2.73 ± 0.02 ^h^	5.09 ± 0.02 ^a^	3.57 ± 0.01 ^c^	1.78 ± 0.01 ^k^	2.41 ± 0.01 ^j^	2.76 ± 0.02 ^g^	nd	3.14 ± 0.01 ^f^	2.41 ± 0.01 ^j^	5.06 ± 0.01 ^b^	3.41 ± 0.03 ^e^	3.52 ± 0.001 ^d^
**Peak** 13	20.2 ± 0.50 ^h^	36.8 ± 0.3 ^b^	30.9 ± 0.5 ^d^	14.89 ± 0.04 ^j^	30.27 ± 0.04 ^e^	30.9 ± 0.8 ^d^	29.8 ± 0.4 ^f^	44.3 ± 0.2 ^a^	34.1 ± 0.5 ^c^	30.6 ± 0.4 ^de^	25.9 ± 0.4 ^g^	16.7 ± 0.5 ^i^	8.7 ± 0.01 ^k^
TPA	50 ± 1 ^h^	81 ± 1 ^b^	81 ± 2 ^b^	40.8 ± 0.4 ^j^	75.4 ± 0.3 ^d^	72.6 ± 0.2 ^e^	66 ± 1 ^f^	113 ± 1 ^a^	77 ± 1 ^c^	76.351 ± 0.003 ^c^	59 ± 1 ^g^	43.97 ± 0.04 ^i^	26.3 ± 0.5 ^k^
TF	17.81 ± 0.01 ^l^	25.0 ± 0.01 ^e^	29.85 ± 0.03 ^a^	28.04 ± 0.04 ^c^	15.86 ± 0.03 ^m^	21.62 ± 0.01 ^j^	23.04 ± 0.01 ^g^	22.988 ± 0.002 ^h^	25.1 ± 0.1 ^d^	21.00 ± 0.02 ^k^	24.86 ± 0.01 ^f^	22.2 ± 0.1 ^i^	29.75 ± 0.02 ^b^
TPC	68 ± 1 ^k^	106 ± 1 ^c^	111 ± 2 ^b^	68.9 ± 0.5 ^i^	91.3 ± 0.3 ^g^	94.2 ± 0.2 ^f^	89 ± 1 ^h^	136 ± 1 ^a^	102 ± 1 ^d^	97.36 ± 0.02 ^e^	84 ± 1 ^i^	66.17 ± 0.02 ^l^	56.1 ± 0.5 ^m^
**Aqueous Extract**													
**Peak** 1	1.18 ± 0.06 ^d^	0.87 ± 0.04 ^g^	1.02 ± 0.02 ^e^	0.59 ± 0.02 ^h^	1.31 ± 0.01 ^b^	1.20 ± 0.06 ^cd^	1.23 ± 0.01 ^c^	7.9 ± 0.1 ^a^	1.19 ± 0.06 ^d^	0.91 ± 0.05 ^fg^	0.96 ± 0.04 ^f^	1.02 ± 0.05 ^e^	0.28 ± 0.04 ^i^
**Peak** 2	2.36 ± 0.02 ^h^	1.97 ± 0.04 ^j^	2.65 ± 0.01 ^f^	2.55 ± 0.08 ^g^	2.31 ± 0.01 ^hi^	3.8 ± 0.1 ^c^	3.83 ± 0.03 ^c^	5.03 ± 0.07 ^a^	2.37 ± 0.05 ^h^	2.28 ± 0.05 ^i^	4.3 ± 0.01 ^b^	3.19 ± 0.03 ^d^	3.06 ± 0.08 ^e^
**Peak** 3	3.5 ± 0.1 ^d^	3.18 ± 0.03 ^g^	3.002 ± 0.002 ^h^	1.52 ± 0.06 ^l^	3.22 ± 0.02 ^f^	3.83 ± 0.02 ^c^	4.19 ± 0.06 ^a^	4.13 ± 0.03 ^b^	2.98 ± 0.04 ^h^	3.30 ± 0.03 ^e^	2.77 ± 0.08 ^j^	2.19 ± 0.09 ^k^	2.90 ± 0.05 ^i^
**Peak** 4	3.25 ± 0.006 ^f^	3.46 ± 0.08 ^e^	2.917 ± 0.004 ^h^	2.80 ± 0.03 ^i^	3.29 ± 0.04 ^f^	3.14 ± 0.08 ^g^	3.99 ± 0.08 ^b^	3.87 ± 0.05 ^c^	3.18 ± 0.04 ^g^	2.80 ± 0.04 ^i^	2.61 ± 0.05 ^j^	3.63 ± 0.01 ^d^	4.35 ± 0.04 ^a^
**Peak** 5	6.49 ± 0.02 ^c^	6.71 ± 0.02 ^b^	8.76 ± 0.01 ^a^	3.681 ± 0.003 ^k^	5.18 ± 0.04 ^f^	5.28 ± 0.01 ^d^	4.1 ± 0.10 ^i^	4.45 ± 0.02 ^g^	3.32 ± 0.02 ^l^	3.79 ± 0.01 ^j^	4.34 ± 0.01 ^h^	2.95 ± 0.03 ^m^	5.22 ± 0.03 ^e^
**Peak** 6	12.83 ± 0.04 ^d^	9.36 ± 0.02 ^h^	8.87 ± 0.03 ^i^	16.73 ± 0.04 ^a^	8.6 ± 0.1 ^k^	10.8 ± 0.1 ^g^	11.6 ± 0.10 ^f^	12.9 ± 0.1 ^d^	8.17 ± 0.02 ^l^	12.97 ± 0.04 ^c^	13.31 ± 0.05 ^b^	8.8 ± 0.1 ^j^	12.26 ± 0.02 ^e^
**Peak** 7	2.52 ± 0.01 ^cd^	2.41 ± 0.09 ^e^	3.33 ± 0.01 ^a^	1.6 ± 0.2 ^h^	2.45 ± 0.08 ^de^	2.26 ± 0.04 ^f^	2.38 ± 0.07 ^e^	2.83 ± 0.01 ^b^	2.61 ± 0.03 ^c^	3.3 ± 0.2 ^a^	2.16 ± 0.09 ^g^	2.74 ± 0.01 ^b^	2.11 ± 0.06 ^g^
**Peak** 8	70.81 ± 0.04 ^g^	64.31 ± 0.02 ^k^	76.4 ± 0.1 ^d^	101.5 ± 0.1 ^a^	42.12 ± 0.01 ^m^	66.8 ± 0.1 ^i^	65.9 ± 0.1 ^j^	84.1 ± 0.1 ^c^	73.56 ± 0.09 ^f^	67.35 ± 0.08 ^h^	73.8 ± 0.1 ^e^	53.6 ± 0.2 ^l^	99.43 ± 0.02 ^b^
**Peak** 9	4.4 ± 0.1 ^ef^	3.26 ± 0.03 ^i^	4.4 ± 0.4 ^ef^	6.6 ± 0.1 ^a^	3.73 ± 0.03 ^h^	4.3 ± 0.5 ^g^	4.6 ± 0.1 ^e^	5.2 ± 0.1 ^d^	6.2 ± 0.5 ^b^	5.7 ± 0.4 ^c^	3.30 ± 0.01 ^i^	4.3 ± 0.30 ^g^	5.8 ± 0.3 ^c^
**Peak** 10	290.0 ± 0.4 ^e^	293 ± 3 ^d^	353.0 ± 0.3 ^b^	190 ± 2 ^j^	288.6 ± 0.2 ^f^	318.0 ± 0.7 ^c^	211 ± 1 ^i^	550 ± 3 ^a^	266.3 ± 0.7 ^g^	291 ± 4 ^e^	218.4 ± 0.5 ^h^	52.2 ± 0.6 ^k^	27.1 ± 0.3 ^l^
**Peak** 11	11.5 ± 0.3 ^i^	13.9 ± 0.4 ^g^	16.37 ± 0.06 ^c^	15.1 ± 0.4 ^d^	9.8 ± 0.3 ^j^	12.2 ± 0.6 ^h^	9.63 ± 0.02 ^j^	16.8 ± 0.2 ^b^	14.83 ± 0.07 ^de^	14.7 ± 0.4 ^e^	14.14 ± 0.09 ^f^	16.9 ± 0.1 ^b^	26.2 ± 0.6 ^a^
**Peak** 12	11.25 ± 0.03 ^e^	7.75 ± 0.02 ^j^	16.08 ± 0.01 ^b^	15.1 ± 0.1 ^c^	4.45 ± 0.04 ^k^	8.54 ± 0.04 ^i^	8.9 ± 0.1 ^h^	nd	10.9 ± 0.1 ^f^	8.51 ± 0.02 ^i^	19.8 ± 0.1 ^a^	9.73 ± 0.02 ^g^	13.7 ± 0.1 ^d^
**Peak** 13	381 ± 5 ^d^	390 ± 2 ^c^	366.7 ± 0.9 ^e^	212.9 ± 0.4 ^k^	309.9 ± 0.4 ^h^	405 ± 3 ^b^	279 ± 2 ^j^	582 ± 1 ^a^	343 ± 2 ^f^	340 ± 4 ^g^	305.7 ± 0.5 ^i^	62.8 ± 0.3 ^l^	47.7 ± 0.8 ^m^
TPA	699 ± 5 ^d^	712 ± 1 ^c^	753.4 ± 0.7 ^b^	433 ± 3 ^j^	621.6 ± 0.8 ^g^	754 ± 3 ^b^	519 ± 3 ^i^	1177 ± 3 ^a^	643 ± 2 ^f^	664 ± 9 ^e^	554.3 ± 0.1 ^h^	148.9 ± 0.6 ^k^	119 ± 1 ^l^
TF	101.4 ± 0.2 ^e^	88.13 ± 0.05 ^j^	110.06 ± 0.01 ^d^	137.0 ± 0.2 ^a^	60.3 ± 0.1 ^l^	91.4 ± 0.1 ^h^	90.4 ± 0.2 ^i^	101.4 ± 0.2 ^e^	96.0 ± 0.2 ^f^	92.6 ± 0.1 ^g^	111.2 ± 0.1 ^c^	75.1 ± 0.3 ^k^	130.6 ± 0.2 ^b^
TPC	801 ± 5 ^d^	801 ± 1 ^d^	863.5 ± 0.7 ^b^	570 ± 3 ^j^	681.9 ± 0.7 ^g^	845 ± 3 ^c^	610 ± 3 ^i^	1278 ± 3 ^a^	739 ± 2 ^f^	756 ± 9 ^e^	665.6 ± 0.2 ^h^	224 ± 1 ^l^	250 ± 1 ^k^

Notes: nd-not detected; tr-traces. TPA—Total phenolic acids; TF—Total flavonoids; TPC—Total phenolic compounds; Peaks: 1—caffeic acid hexoside; 2—caffeic acid hexoside; 3—*p*-coumaroyl hexoside; 4—caffeic acid; 5—luteolin-*O*-hexosyl-*O*-glucuronide; 6—eriodictyol-*O*-glucuronide; 7—rosmarinic acid hexoside; 8—luteolin-7-*O*-glucuronide; 9—sangerinic acid; 10—rosmarinic acid; 11—lithospermic acid A; 12—methylluteolin-*O*-glucuronide; 13—salvianolic acid B. Standard calibration curves: caffeic acid (y = 388,345x + 406,369, R² = 0.994; LOD = 0.78 μg/mL; LOQ = 1.97 μg/mL) (compounds 1, 2 and 4); *p*-coumaric acid (y = 301,950x + 6966.7, R² = 0.999; LOD = 0.68 μg/mL; LOQ = 1.61 μg/mL) (compound **3**); luteolin-7-*O*-glucoside (y = 4087x + 72,589, R² = 0.999; LOD = 0.21 μg/mL; LOQ = 0.74 μg/mL) (compounds **5**, **8** and **12**); hesperetin (y = 34156x + 268,027, R² = 0.999; LOD = 0.42 µg/mL; LOQ = 0.87 µg/mL) (compound **6**); and rosmarinic acid (y = 191,291x − 652,903, R² = 0.999; LOD = 0.15 µg/mL; LOQ = 0.68 µg/mL) (compounds **7**, **9**, **10**, **11** and **13**). The results were analyzed using one-way analysis of variance (ANOVA) followed by Tukey’s HSD Test, and in each row and for each extract (hydroethanolic or aqueous extracts) different letters (a to m) mean significant differences among total compounds (*p* < 0.05).

**Table 3:** Antioxidant activity and anti-inflammatory potential of *L. pedunculata* hydroethanolic and aqueous extracts (mean ± SD).

	1	2	3	4	5	6	7	8	9	10	11	12	13
**Hydroethanolic Extracts**													
**Antioxidant Activity (EC_50_ Values, μg/mL)**													
DPPH scavenging activity	142 ± 6 ^ef^	139 ± 3 ^ef^	139 ± 2 ^ef^	212 ± 8 ^b^	137 ± 3 ^f^	139 ± 1 ^f^	150 ± 3 ^d^	87 ± 2 ^g^	150 ± 2 ^d^	146 ± 8 ^de^	181 ± 2 ^c^	140 ± 5 ^ef^	257 ± 7 ^a^
Reducing power	110 ± 1 ^f^	98 ± 1 ^g^	117.9 ± 0.5 ^e^	149 ± 2 ^b^	97 ± 2 ^g^	107 ± 2 ^f^	129 ± 1 ^d^	72 ± 1 ^h^	67 ± 1 ^i^	135 ± 9 ^c^	133 ± 3 ^cd^	133 ± 1 ^cd^	216 ± 6 ^a^
β-carotene bleaching inhibition	1009 ± 88 ^d^	821 ± 34 ^e^	1062 ± 65 ^d^	516 ± 75 ^f^	1176 ± 40 ^c^	1578 ± 77 ^b^	541 ± 43 ^f^	1833 ± 178 ^a^	372 ± 18 ^g^	252 ± 7 ^h^	190 ± 20 ^h^	223 ± 3 ^h^	214 ± 10 ^h^
TBARS inhibition	28 ± 1 ^g^	17 ± 1 ^i^	26 ± 1 ^h^	35 ± 1 ^e^	38.25 ± 0.01 ^d^	63.5 ± 0.1 ^a^	44.56 ± 0.01 ^c^	25.57 ± 0.02 ^h^	38.2 ± 0.3 ^d^	48 ± 2 ^b^	62 ± 4 ^a^	27.7 ± 0.05 ^gh^	31.4 ± 0.1 ^f^
**Anti-Inflammatory Potential (EC_50_ Values, µg/mL)**													
Nitric oxide (NO) production	190 ± 3 ^c^	198 ± 5 ^b^	190 ± 6 ^c^	>400	>400	124 ± 8 ^f^	>400	>400	>400	216 ± 12 ^a^	171 ± 3 ^d^	162 ± 4 ^e^	>400
**Aqueous Extracts**													
**Antioxidant Activity (EC_50_ Values. μg/mL)**													
DPPH scavenging activity	109 ± 4 ^f^	99 ± 2 ^g^	99 ± 1 ^g^	68.0 ± 0.5 ^i^	99 ± 2 ^g^	115 ± 1 ^e^	125 ± 2 ^d^	69 ± 3 ^h^	98 ± 3 ^g^	115 ± 2 ^e^	133 ± 3 ^c^	144 ± 6 ^b^	191 ± 2 ^a^
Reducing power	101 ± 2 ^f^	51 ± 1 ^i^	113 ± 4 ^e^	137 ± 4 ^b^	99 ± 2 ^f^	93 ± 3 ^g^	111 ± 2 ^e^	73 ± 1 ^h^	110 ± 2 ^e^	125 ± 4 ^cd^	122 ± 6 ^d^	130 ± 6 ^c^	167 ± 1 ^a^
β-carotene bleaching inhibition	274 ± 10 ^f^	253 ± 14 ^fg^	267 ± 9 ^f^	385 ± 13 ^d^	472 ± 14 ^b^	475 ± 19 ^b^	422 ± 25 ^c^	388 ± 21 ^d^	452 ± 12 ^b^	236 ± 14 ^g^	305 ± 4 ^e^	391 ± 25 ^d^	530 ± 21 ^a^
TBARS inhibition	14 ± 1 ^i^	24.8 ± 0.4 ^de^	16 ± 1 ^h^	29 ± 1 ^c^	21.8 ± 0.4 ^fg^	22 ± 1 ^f^	21.15 ± 0.01 ^g^	21.2 ± 0.2 ^g^	21 ± 1 ^g^	39.1 ± 0.1 ^a^	25 ± 1 ^d^	24.1 ± 0.4 ^e^	36 ± 1 ^b^
**Anti-Inflammatory Potential (EC_50_ Values. µg/mL)**													
Nitric oxide (NO) production	>400	>400	>400	301 ± 7 ^a^	237 ± 7 ^b^	>400	205 ± 7 ^c^	140 ± 5 ^d^	>400	>400	>400	>400	>400

Notes: EC_50_: Extract concentration corresponding to 50% of antioxidant activity or 0.5 of absorbance in reducing power assay. Trolox (positive control) EC_50_ values: 41 µg/mL (reducing power), 42 µg/mL (DPPH scavenging activity), 18 µg/mL (β-carotene bleaching inhibition) and 23 µg/mL (TBARS inhibition). Anti-inflammatory activity is expressed as EC_50_ values corresponding to 50% of inhibition of the NO production in comparison with the negative control (100% of NO production). Dexamethasone (positive control) EC_50_ values: 16 µg/mL. The results were analyzed using one-way analysis of variance (ANOVA) followed by Tukey’s HSD Test, and in each row different letters (a to i) mean significant differences (*p* < 0.05).

**Table 4:** Cytotoxic properties of *L. pedunculata* hydroethanolic and aqueous extracts (mean ± SD).

	1	2	3	4	5	6	7	8	9	10	11	12	13
**Hydroethanolic Extracts**													
**Tumor Cell Lines (GI_50_ Values, µg/mL)**													
MCF-7	61 ± 2 ^f^	57 ± 3 ^fg^	58 ± 2 ^fg^	115 ± 9 ^c^	82 ± 8 ^d^	53 ± 5 ^g^	70 ± 2 ^e^	61 ± 4 ^f^	60 ± 4 ^f^	53 ± 3 ^g^	61 ± 3 ^f^	236 ± 8 ^a^	212 ± 1 ^b^
NCI-H460	285 ± 22 ^bc^	275 ± 22 ^c^	277 ± 10 ^c^	>400	>400	340 ± 21 ^a^	>400	>400	>400	>400	297 ± 6 ^b^	119 ± 7 ^e^	241 ± 23 ^d^
HeLa	70 ± 5 ^de^	65.0 ± 0.2 ^fgh^	69 ± 2 ^def^	200 ± 14 ^c^	216 ± 7 ^b^	66.2 ± 0.2 ^efgh^	67 ± 2 ^efgh^	63.3 ± 0.1 ^gh^	73.1 ± 0.3 ^d^	62.2 ± 0.6 ^h^	65 ± 2 ^fgh^	68 ± 3 ^efg^	222 ± 6 ^a^
HepG2	82 ± 4 ^e^	67 ± 1 ^f^	94 ± 6 ^d^	144 ± 10 ^c^	191 ± 16 ^b^	34 ± 3 ^g^	212 ± 19 ^a^	67 ± 10 ^f^	100 ± 3 ^d^	62 ± 5 ^f^	65 ± 1 ^f^	203 ± 14 ^a^	204 ± 4 ^a^
**Non-Tumor Cells (GI_50_ Values, µg/mL)**													
PLP2	>400	>400	>400	>400	>400	>400	>400	>400	>400	>400	>400	291 ± 11	>400
**Aqueous Extracts**													
**Tumor Cell Lines (GI_50_ Values, µg/mL)**													
MCF-7	>400	270 ± 14 ^c^	267 ± 2 ^c^	287 ± 8 ^b^	267.7 ± 0.3 ^c^	222 ± 14 ^e^	223 ± 6 ^e^	185 ± 16 ^g^	150 ± 9 ^h^	290 ± 12 ^b^	256.2 ± 0.6 ^d^	270 ± 14 ^f^	342 ± 14 ^a^
NCI-H460	329 ± 15 ^c^	256 ± 5 ^e^	334 ± 15 ^c^	113 ± 9 ^i^	142 ± 12 ^h^	226 ± 14 ^f^	188 ± 12 ^g^	183 ± 11 ^g^	258 ± 14 ^e^	245 ± 13 ^e^	349 ± 5 ^b^	293 ± 12 ^d^	374 ± 7 ^a^
HeLa	>400	>400	310.2 ± 0.4 ^b^	343.92 ± 0.01 ^a^	253 ± 23 ^d^	262 ± 14 ^d^	199 ± 12 ^f^	159 ± 16 ^g^	286 ± 4 ^c^	195 ± 11 ^f^	224 ± 11 ^e^	82 ± 14 ^h^	298 ± 17 ^bc^
HepG2	315 ± 6 ^a^	254 ± 14 ^c^	251 ± 14 ^c^	293 ± 10 ^b^	>400	211 ± 17 ^d^	194 ± 15 ^e^	179 ± 14 ^f^	118 ± 8 ^h^	256 ± 4 ^c^	191 ± 143 ^ef^	148 ± 1 ^g^	324 ± 6 ^a^
**Non-Tumor Cells (GI_50_ Values, µg/mL)**													
PLP2	>400	>400	>400	>400	>400	>400	349 ± 12 ^b^	362 ± 21 ^a^	240 ± 16 ^c^	>400	>400	>400	>400

MCF-7: breast carcinoma; NCI-H460: non-small lung cancer; HeLa: cervical carcinoma; HepG2: hepatocellular carcinoma; GI50 values—concentration that inhibited 50% of the net cell growth. Ellipticine (positive control) GI_50_ values: 1.21 µg/mL (MCF-7), 1.03 µg/mL (NCI-H460), 0.91 µg/mL (HeLa), 1.10 µg/mL (HepG2) and 2.29 µg/mL (PLP2). The results were analyzed using one-way analysis of variance (ANOVA) followed by Tukey’s HSD Test, and in each row different letters (a to h) mean significant differences (p < 0.05).

**Table 5:** Antibacterial (MIC and MBC, μg/mL) and antifungal (MIC and MFC, μg/mL) activities of *L. pedunculata* hydroethanolic and aqueous extracts.

**Antibacterial Activity**															
**Hydroethanolic Extracts**															
	1	2	3	4	5	6	7	8	9	10	11	12	13	S	A
	MIC	MIC	MIC	MIC	MIC	MIC	MIC	MIC	MIC	MIC	MIC	MIC	MIC	MIC	MIC
	MBC	MBC	MBC	MBC	MBC	MBC	MBC	MBC	MBC	MBC	MBC	MBC	MBC	MBC	MBC
B.c.	50	75	75	75	50	75	75	75	75	75	75	75	200	100	250
	75	150	150	150	75	150	150	150	150	150	150	150	300	200	400
M.f.	75	100	100	100	75	100	100	100	100	100	100	300	200	200	250
	150	150	150	150	150	150	150	150	150	150	150	450	300	300	400
S.a.	20	40	30	150	100	150	150	150	200	150	200	450	200	40	250
	40	75	40	300	150	300	300	300	150	300	300	600	300	100	450
L.m.	100	150	100	150	100	150	150	150	150	150	200	450	200	200	400
	150	300	150	300	150	300	300	300	300	300	300	600	300	300	500
E.c.	75	100	100	100	75	100	100	100	100	100	100	200	450	200	400
	150	150	150	150	150	150	150	150	150	150	150	300	600	300	500
En.cl.	40	40	40	50	40	40	100	75	75	75	75	200	450	200	250
	75	75	75	75	75	75	150	150	150	150	150	300	600	300	500
P.a.	100	150	100	150	100	150	150	150	150	150	150	300	150	200	750
	150	300	150	300	150	300	300	300	300	300	300	450	300	300	1200
S.t.	150	150	150	200	100	200	200	150	200	200	200	150	150	250	400
	300	300	300	300	150	300	300	300	300	300	300	300	300	500	750
**Aqueous Extracts**															
	1	2	3	4	5	6	7	8	9	10	11	12	13	S	A
	MIC	MIC	MIC	MIC	MIC	MIC	MIC	MIC	MIC	MIC	MIC	MIC	MIC	MIC	MIC
	MBC	MBC	MBC	MBC	MBC	MBC	MBC	MBC	MBC	MBC	MBC	MBC	MBC	MBC	MBC
B.c.	200	300	150	300	75	200	150	200	150	150	150	300	150	100	250
	300	450	300	450	150	300	300	300	300	300	300	450	300	200	400
M.f.	200	300	200	300	200	300	200	200	200	150	200	450	200	200	250
	300	450	300	450	300	450	300	300	300	300	300	600	300	300	400
S.a.	300	300	300	300	450	300	200	200	150	300	300	300	300	40	250
	450	450	600	450	600	450	300	300	300	450	450	600	600	100	450
L.m.	300	300	300	300	450	300	300	300	150	300	300	300	300	200	400
	600	600	600	600	600	450	450	450	300	450	450	600	600	300	500
E.c.	200	200	200	200	200	200	200	100	200	450	200	450	200	200	40
	300	300	300	300	300	300	300	150	300	600	300	900	300	300	500
En.cl.	150	200	200	200	200	150	200	150	150	200	150	200	150	200	250
	300	300	300	300	300	300	300	300	300	300	300	300	300	300	500
P.a.	300	450	300	300	300	300	300	150	150	300	300	300	300	200	750
	450	600	600	600	600	450	600	450	300	450	600	600	600	300	1200
S.t.	450	450	300	300	300	300	300	300	150	300	300	300	300	250	400
	600	600	600	600	600	600	600	450	300	450	600	600	600	500	750
**Antifungal Activity**															
**Hydroethanolic Extracts**															
	1	2	3	4	5	6	7	8	9	10	11	12	13	K	B
	MIC	MIC	MIC	MIC	MIC	MIC	MIC	MIC	MIC	MIC	MIC	MIC	MIC	MIC	MIC
	MFC	MFC	MFC	MFC	MFC	MFC	MFC	MFC	MFC	MFC	MFC	MFC	MFC	MFC	MFC
A.fum.	75	150	75	75	100	40	150	75	100	150	150	200	200	250	150
	150	300	150	150	150	75	300	150	150	300	300	300	300	500	200
A.v.	40	30	40	40	75	40	40	40	75	150	75	150	150	200	100
	75	75	150	75	150	75	75	75	150	300	150	300	300	500	200
A.o.	40	40	50	40	75	40	50	40	20	75	40	100	200	150	150
	75	75	75	75	150	75	75	100	40	150	75	300	300	2000	200
A.n.	50	75	40	75	150	75	150	75	150	150	200	150	200	200	150
	75	150	75	150	300	150	300	150	300	300	300	300	300	500	200
T.v.	20	40	20	30	75	30	30	50	15	50	30	75	100	1000	150
	40	75	40	40	150	40	40	75	20	75	40	150	150	1000	200
P.f.	75	75	40	75	200	75	75	40	75	75	150	200	200	200	200
	150	150	75	150	300	15	150	75	150	150	300	300	300	500	250
P.o.	40	40	30	40	100	40	40	150	40	75	75	30	30	2500	200
	75	75	40	75	150	75	75	200	75	150	150	450	450	3500	250
P.v.c.	75	150	50	100	200	75	150	150	150	150	150	300	300	200	100
	150	300	75	150	300	150	300	300	300	300	300	450	450	300	200
**Aqueous Extracts**															
	1	2	3	4	5	6	7	8	9	10	11	12	13	K	B
	MIC	MIC	MIC	MIC	MIC	MIC	MIC	MIC	MIC	MIC	MIC	MIC	MIC	MIC	MIC
	MFC	MFC	MFC	MFC	MFC	MFC	MFC	MFC	MFC	MFC	MFC	MFC	MFC	MFC	MFC
A.fum.	300	300	600	-	600	300	-	-	600	600	300	300	450	250	150
	600	600	1200	-	1200	600	-	-	900	1200	450	600	900	500	200
A.v.	300	300	300	600	300	300	300	300	300	200	100	150	200	200	100
	600	600	600	1200	600	600	600	600	600	450	200	300	300	500	200
A.o.	600	900	-	-	-	600	300	-	200	300	150	300	200	1500	150
	1200	1200	-	-	-	1200	600	-	450	600	300	450	450	2000	200
A.n.	-	300	-	-	-	600	-	-	300	-	200	450	450	200	150
	-	600	-	-	-	1200	-	-	450	-	450	900	900	500	200
T.v.	600	300	600	600	-	600	-	-	100	300	200	450	450	1000	150
	1200	600	1200	1200	-	1200	-	-	300	600	450	600	600	1000	200
P.f.	600	300	600	-	600	-	-	-	-	-	450	450	450	200	200
	1200	600	1200	-	1200	-	-	-	-	-	600	900	900	500	250
P.o.	900	450	600	-	600	-	-	-	200	-	450	450	450	2500	200
	1200	600	1200	-	1200	-	-	-	450	-	600	900	900	3.50	0.25
P.v.c.	-	600	600	-	-	-	-	-	200	450	450	450	450	200	100
	-	1200	1200	-	-	-	-	-	450	900	600	900	900	300	200

Notes: MIC: Minimal Inhibitory Concentration; MBC: Minimal Bactericidal Concentration; MFC: Minimal Fungicide Concentration; S: Streptomycin; A: Ampicillin; K: Ketoconazole; B: Bifonazole; B.c.: *Bacillus cereus; M.f.: Micrococcus flavus; S.a.: Staphylococcus aureus; L.m.: Listeria monocytogenes; E.c.: Escherichia coli; En. cl.: Enterobacter cloacae; P.a.: Pseudomonas aeruginosa; S.t.: Salmonella typhimirium; A.fum.: Aspergillus fumigatus; A.v.: Aspergillus versicolor; A.o.: Aspergillus ochraceus; A.n.: Aspergillus niger; T.v.: Trichoderma viride; P.f.: Penicillium funiculosum; P.o.: Penicillium ochrochloron; P.v.c.: Penicillium verrucosum var. cyclopium.*

**Table 6:** Geographical information about the studied *L. pedunculata* samples.

Samples	BPGV Accession Number	Origin	Site Data Altitude (m)
1	10378	Marvão. Portalegre	333
2	09845	Évora	198
3	09838	Vila Viçosa. Évora	420
4	11290	Bragança	810
5	10372	Arronches. Portalegre	375
6	10400	Portalegre	375
7	10418	Nisa. Portalegre	291
8	10387	Ponte de Sôr. Portalegre	175
9	10391	Évora	155
10	10412	Castelo de Vide. Portalegre	358
11	10369	Elvas. Portalegre	267
12	10379	Castelo de Vide. Portalegre	353
13	11308	Bragança	667

More information in Lopes and Barata [9]; Accessions passport data documented and available at Grin-Global database (http://bpgv.iniav.pt).
